# Vertiginous dizziness: A primary care approach

**DOI:** 10.4102/safp.v65i1.5712

**Published:** 2023-04-21

**Authors:** Shane D. Murphy, Michael G. van Aardt

**Affiliations:** 1 Department of Clinical Services, Abbey House Medical Centre, Navan, Ireland; 2 Department of Otorhinolaryngology, Faculty of Health Sciences, University of the Witwatersrand, Johannesburg, South Africa

**Keywords:** family medicine, general practice, primary care, vertigo, dizziness

## Abstract

Dizziness is an extremely common, yet complex neurological symptom that reflects a disturbance of normal balance perception and spatial orientation. Dizziness is a non-specific, catch-all term commonly used by patients to describe a wide array of symptoms, including a sensation of motion, weakness, light-headedness, unsteadiness, emotional upset and depression. The national 1-year prevalence of dizziness is around 50%, accounting for 4% of emergency department presentations and 1% of primary care consultations in South Africa. This article will focus on a diagnostic approach to the most common cause of dizziness (vertigo).

## Vertigo

Vertigo is commonly described as the illusion of movement. The illusion of movement could be perceived by patients as either self-motion, or motion of the surrounding environment around them.^1^ Vertigo often poses a diagnostic dilemma as it is a symptom of a host of diagnoses from benign to immediately life-threatening conditions.

The basic pathophysiology of vertigo is that of asymmetry in the vestibular system caused by either dysfunction or damage to the vestibular structures.^2^

**FIGURE 1:**
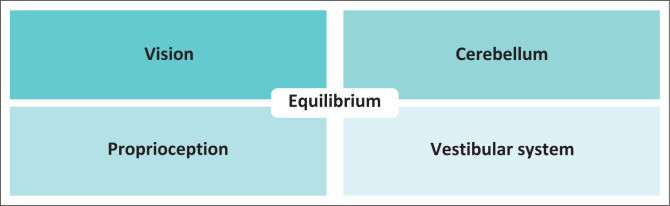
Systems responsible for regulating balance (equilibrium).

The vestibular system is a sensory system responsible for the regulation of balance, oculomotor control, spatial orientation and perception of self-motion.^2^ The vestibular system works in conjunction with vision, the proprioceptive system and the cerebellum to maintain equilibrium (Figure 1). A loss (or mismatch) of any of these can cause the perception of vertigo.

### Pathophysiology

Vestibular labyrinths on each side of the body, consisting of semi-circular canals and an otolith system, transmit signals to the central nervous system (CNS). The CNS then compares these signals to recognise motion – when the head is still, tonic discharges from both sides are balanced.^2^ During movement, the left and right labyrinths are either inhibited or excited, creating a left-right difference in eighth nerve activity. These signals are transmitted to the vestibular nuclei in the brainstem (below the pons and anterior to the cerebellum) with projections to the cerebellum, oculomotor nuclei and spinal cord.^2^ Vestibulo-ocular connections are responsible for coordinated eye movements during head movement, vestibulospinal pathways facilitate upright posture and cerebellar connections modulate these activities. Any dysfunction or disease process in this system produces vertigo.

### Causes of vertigo

It is useful to categorise the causes of vertigo into peripheral and central disorders as they have distinctive clinical features on history and physical examination (Table 1).^1,2,3,4^

**TABLE 1:** Features of peripheral and central vertigo.

Feature	Peripheral	Central
Onset	Sudden	Sudden or gradual
Severity	Initially severe, often subsiding over time	Mild in most cases, can be more severe in stroke and multiple sclerosis
Pattern	Paroxysmal, intermittent episodes last seconds to less than 1 min (BPPV); continuous lasting hours to days (vestibular neuritis)	Constant, usually lasting several weeks Shorter episodes of minutes to hours in vascular causes such as transient ischemic attacks (posterior circulation)
Aggravated by position and/or movement	Yes Either induces (BPPV) or worsens (vestibular neuritis) vertigo	Variable – Minimal change but can worsen with head movement
Nausea and/or diaphoresis	Frequent	Variable
Nystagmus	Usually torsional and upbeat (fast phase beating toward forehead) in classic posterior canal BPPV; horizontal canal BPPV will be horizontal vertigo; horizontal torsional with vestibular neuritis and labyrinthitis Suppressed by visual fixation	Vertical and/or multidirectional; Purely vertical, spontaneous, purely torsional direction-changing on lateral gaze, down beating (fast phase beats towards the nose) Not suppressed by visual fixation
Fatigue of symptoms and/or signs	Yes Tinnitus (Meniere’s disease) and hearing loss (labyrinthitis)	No (rare)
Hearing loss and/or tinnitus	May occur	Seldomly occurs – not a primary feature
Abnormal tympanic membrane	May occur	Seldomly occurs – not a primary feature
Postural instability	Less pronounced. Usually unidirectional instability with preserved walking	Severe instability – patient often falls when walking
CNS symptoms and/or signs	Absent	Usually present Diplopia, ataxia, dysarthria dysphasia, focal or lateralised weakness

*Source:* Please see the full reference list of the article Vertigo – Diagnosis and management in primary care. Br J Med Pract [serial online]. [cited 2022 Dec 13]. Available from: https://www.bjmp.org/content/vertigo-diagnosis-and-management-primary-care, for more information

CNS, central nervous system; BPPV, benign paroxysal positional vertigo.

## Taking a history: Clinical features of vertigo

The spinning sensation of vertigo is notoriously unreliable, with myriad subjective experiences, as well as descriptions thereof. The most common experience is that of spinning, but other descriptions include: ‘whirling’, ‘tilting’, ‘moving’, ‘imbalance’ or ‘disorientation’.^2^

The nondescript nature of vertigo means that the clinician should seek out the clinical features of the presentation alongside associated complaints. Descriptive elements such as the timing (onset, duration and disease course) and triggers (provoking and aggravating factors) are more useful in determining the underlying disease.

### Time course

Carefully establishing the time course is the first step in hypothetico-deductive reasoning and pragmatic classification of vestibular syndrome (see syndromic approach to vertigo; Table 2)^2,3,4^.Vertigo is never continuous for more than a few weeks (although it may be episodic). Even if a permanent vestibular lesion exists, the CNS (other components of equilibrium – see Figure 1) adapts to accommodate for the defect.^2^ In situations where a patient has vertigo for longer than a week, the clinician should clearly establish whether the patient was actually spinning the entire time (if true, points more to central causes), or was having episodic spinning followed by imbalance.

**TABLE 2:** Common conditions in the vestibular syndromes.

Feature	Pathophysiology	Suggestive clinical features	Other diagnostic features
Acute vestibular syndrome			
Vestibular neuritis (Vestibular neuronitis and labyrinthitis)	Viral or post viral inflammation of the vestibular portion of the 8th cranial nerve	Acute onset, single episode lasting several days. Viral syndrome – preceding or accompanying.	Falls towards the side of the lesion. No brainstem features. Head impulse test usually abnormal. Unilateral hearing loss with labyrinthitis. pain on tragal stimulation suggestive of suppurative labyrinthitis (medical emergency).
Posterior circulation stroke	Atherosclerotic or lipo-hyalinotic occlusion of vessels of the posterior cerebral circulation	Acute onset with persistent symptoms (days to weeks). Pronounced gait instability with cerebellar features. More common in older persons with cardiovascular risk factors. Patient falls towards side of the lesion and nystagmus is more pronounced on the side of the lesion in contrast to peripheral lesions where patients fall towards the side of the lesion and nystagmus fast phase is away from the lesion.	Other signs correlate to site of lesion (e.g. Wallenburg syndrome). Urgent Neuroimaging required.
Triggered-episodic vestibular syndrome			
Benign paroxysmal positional vertigo (rarely, central positional paroxysmal vertigo)	Calcium debris within the semi-circular canals (most commonly in posterior)	Repeated, brief spinning sensation (seconds to minutes) with predictable provocation on turning or tilting the head. Mild nausea, rarely vomiting.	Positive Dix-Hallpike (posterior) or supine roll test (horizontal canal).
Spontaneous episodic vestibular syndrome			
Meniere disease	Endolymphatic hydrops excess endolymphatic fluid pressure with consequent inner ear dysfunction Severe, spontaneous recurrent episodes lasting minutes to hours	Falls towards the side of the lesion. No brainstem features. Head impulse test usually abnormal Unilateral hearing loss.	Associated ear fullness/ pain; unilateral hearing loss. Webber and Rinne tests show unilateral sensorineural hearing loss.
Vestibular migraine	Incompletely understood, pathophysiology likely similar to migraine	Recurrent episodes lasting several hours. May have central or peripheral features. Associated history or features of migrainous headaches.	Exam and tests are normal.
Vertebrobasilar ischaemia or transient ischaemic attack	Athero-embolic occlusions of the arterial system	Single or recurrent episodes lasting several minutes to hours. Usually, other brainstem features. More common in older persons with cardiovascular risk factors.	Require neuroimaging and further workup for TIA.
Cerebellar infarction or haemorrhage	Atherosclerotic or lipohyalinotic	Acute onset with persistent symptoms (days to weeks). Pronounced gait instability with cerebellar features. More common in older persons with cardiovascular risk factors falls towards side of the lesion and nystagmus more pronounced on the side of the lesion in contrast to peripheral lesions with patients fall toward the side of the lesion and the nystagmus fast phase is away from the lesion.	Urgent neuroimaging required.

*Source:* Please see the full reference list of the article Schaider JJ, Hayden SR, Wolfe RE, Barkin AZ, Shayne P, Rosen P. Rosen & Barkin’s 5-minute emergency medicine consult [homepage on the Internet]. 6th ed. Wolters Kluwer Health; 2019 [cited 01 Oct 2023]. Available from: https://www.wolterskluwer.com/en/solutions/ovid/rosen--barkins-5minute-emergency-medicine-consult-681, for more information

TIA, transient ischaemic attack.

### Triggers and aggravators

At the outset, it is important to differentiate between postural presyncope and positional vertigo – both associated with dizziness upon standing: determine if the dizziness can be provoked when changing position of the head without lowering blood pressure (such as lying down, looking up and rolling over). All causes of vertigo are made worse by head motion.^3^ If this does not aggravate the dizziness, it is probably not vertigo.^4^

### Nausea and vomiting

Nausea and vomiting are typical features of acute vertigo, more common (and pronounced) in peripheral causes.^3^ The severity ranges from mild/brief (BPPV) to severe (such as Meniere’s disease), potentially causing dehydration and electrolyte imbalance.

### Nystagmus

The presence of nystagmus (although not always readily visible) is strongly suggestive of vertigo.^2^ Some types of nystagmus are only seen after provocative manoeuvres (such as the Dix-Hallpike Manoeuvre or the lateral gaze test). Additionally, nystagmus can be unmasked in peripheral vertigo when you remove visual fixation (e.g. when wearing Frenzel lenses). The characteristics of the nystagmus can assist with differentiating peripheral from central causes (see Table 1).^1,2,3,4^

### Postural and gait instability

Postural and gait instability can be found in several causes of vertigo. Central causes, however, produce more pronounced symptoms (the vestibulospinal tract receives signals from the vestibular nuclei and in turn stimulates antigravity muscles for the maintenance of posture).^5^

### Other features

Other, less reliable features of vestibular dysfunction include:^2^

- Tilt illusion
  ■ Patients feel as if they and/or their environments are tilted with respect to gravity.
  ■ Suggestive of otolith system dysfunction.
- Drop attacks
  ■ Described as a sensation of being pushed or pulled to the ground. Not associated with faintness (presyncope) or loss of consciousness (seizures).
- Oscillopsia
  ■ Illusory to-and-fro motion of the environment with associated blurred vision when there is movement of the head. Patients often have to stop to read signs, and others.
  ■ If it happens during movement (head movement; walking; driving; head shaking) then it is likely peripheral pathology.
  ■ If it happens in certain head positions (e.g. when supine but not when seated) or if unrelated to movement, it reflects a positional nystagmus/acquired CNS nystagmus – strongly suggestive of an impaired vestibulo-ocular reflex.
- Impaired balance without vertigo
  ■ If there is bilateral vestibular dysfunction, vestibular asymmetry is not pronounced and no vertigo occurs.
  ■ Aminoglycoside toxicity is the most common cause, but this can occur in Meniere’s disease, meningitis, midline cerebellar lesions and thiamine deficiency (Wernicke’s encephalopathy).

## A syndromic approach

Start by taking a standard history to identify and/or rule out toxic, metabolic and infectious causes.

*Patient profile and risk factors play an important role in reaching an accurate diagnosis and informing your index of suspicion (i.e. elderly, hypertensive and smokers raise the suspicion of thromboembolic central causes, while a younger female with a history of migraines would be more indicative of vestibular migraine)*

Use a syndromic approach (of three vestibular syndromes – Box 1) when taking a history.^4^ This facilitates targeted hypothetico-deductive reasoning using timing and triggers (see clinical features) to categorise the type of vertigo.

BOX 1 Vertigo syndromes.

**Acute vestibular syndrome (AVS)**

- Acute onset of continuous dizziness (> 24h)
- Motion intolerance, nausea, and (often) vomiting and (sometimes) gait instability
- Motion will exacerbate the dizziness, but the dizziness is always present
- Causes:
  ■ Most common - vestibular neuritis and labyrinthitis
  ■ Other - stroke (posterior circulation), Multiple sclerosis, thiamine deficiency (Wernicke’s encephalopathy)

**Triggered episodic vestibular syndrome (t-EVS)**

- Usually intermittent, brief episodes
- asymptomatic between episodes
- obligate trigger (e.g. when turning head)
- Causes:
  ■ Commonly BPPV
  ■ Other: Orthostatic hypotension, central positional paroxysmal vertigo (rare)

**Spontaneous-episodic vestibuter syndrome (s-EVS)**

- Usually longer episodes of dizziness
- No identifiable trigger
- Causes:
  ■ Vestibular:Vestibular migraine, Transient Ischaemic Attack (posterior circulation). Meniere disease
  ■ Non-vestibular: Panic attacks, rarely, intermittent low flow states (e.g. dysrhythmias or PE)

*Source:* Adapted from Vertigo – Diagnosis and management in primary care. Br J Med Pract [serial online]. [cited 2022 Dec 13]. Available from: https://www.bjmp.org/content/vertigo-diagnosis-and-management-primary-care

## Physical examination

The physical examination is informed by the classification of the presentation into each of the vestibular syndromes.

### Acute vestibular syndrome (AVS)

- Perform a head impulse, nystagmus, and test of skew (HINTS) exam: head impulse test (HIT), nystagmus testing, and testing for skew deviation (alternate cover test). The HINTS exam consists of three tests and helps to differentiate between central and peripheral causes of acute persistent vertigo:^6^
  ■ First test for nystagmus (most patients with AVS will have nystagmus and its characteristics are important): (1) direction-fixed nystagmus is often peripheral in origin; (2) direction-changing, vertical, or primarily torsional nystagmus is likely of central origin; (3) If there is no nystagmus, vestibular neuritis or labyrinthitis is highly unlikely - in this case, testing the gait is important, while the HIT is less useful.
  ■ [spaced endash after unlikely]
  ■ Perform the alternate cover test to look for skew deviation (see method below): (1) Realignment of the eye vertically is suggestive of a central pathology; (2) No realignment is suggestive of a peripheral cause.
  ■ If nystagmus was present, perform the HIT: (1) A corrective saccade (horizontal eye movement) suggestive of a peripheral cause (e.g. vestibular neuritis); (2) The absence of a corrective saccade is consistent with a central cause (such as a posterior circulation infarct).
- Test the cranial nerves
  ■ Look for any abnormality, implying a structural diagnosis in the posterior fossa
- Test cerebellar function
  ■ Finger-to-nose and heel-to-shin testing
    ◦ Suggestive of midline and/or posterior fossa involvement
- Finally, test gait:
  ■ Most patients with vestibular neuritis can ambulate, while most with a central cause cannot
  ■ If the patient is too nauseous to walk, test for truncal ataxia (strongly suggestive of a central cause)

### Triggered-episodic vestibular syndrome (t-EVS)^6^

- Measure orthostatic vital signs (if postural hypotension is suspected)
- If BPPV is likely diagnosis:
  ■ Perform the Dix-Hallpike manoeuvre bilaterally (posterior semicircular canal)
  ■ Dix-Hallpike will be positive on one side (provoked or triggered symptoms, and usually up-beating torsional nystagmus) and negative on the other side
  ■ If Dix-Hallpike is bilaterally negative, test the horizontal canals by doing the supine head roll test – in the supine head roll, both sides will be positive but one will be much more intense.

### Spontaneous-episodic vestibular syndrome (s-EVS)

- By definition, the patient is asymptomatic, and dizziness cannot be triggered at the bedside.^4^

## Workup

Further workup and/or the need for referral is guided by the syndromic categorisation and suspected disease process.

In AVS, patients with characteristic nystagmus, absent skew deviation, a normal HIT, and a normal neurologic exam (including, cranial nerve, cerebellar and gait testing) need no further workup and can be treated for vestibular neuritis and/or labyrinthitis.^4^

In t-EVS, patients with a positive positional manoeuvre (Dix-Hallpike or Supine roll) do not need any workup and can be treated for BPPV (Epley manoeuvre or Gufoni manoeuvre). Patients with t-EVS who are orthostatic need referral for testing for the cause of the orthostasis (dysautonomia, postural orthostatic tachycardia syndrome, etc.).

Patients with spontaneous episodic vestibular syndrome (s-EVS) need a TIA workup, unless vestibular migraine is likely, and/or previously diagnosed.^1,3^
